# Benchmarking hospital safety and identifying determinants of hospital-acquired complication: the case of Queensland cardiac linkage longitudinal cohort

**DOI:** 10.1016/j.infpip.2021.100198

**Published:** 2021-12-13

**Authors:** Son Nghiem, Clifford Afoakwah, Paul Scuffham, Joshua Byrnes

**Affiliations:** a Centre for Applied Health Economics, School of Medicine and Dentistry, Griffith University, 170 Kessels Rd, Nathan, QLD 4111, Australia; b Menzies Health Institute Queensland, G40, Gold Coast Campus, Griffith University QLD 4222, Australia

**Keywords:** Hospital-acquired complications, Cardiovascular disease, Linked administrative health data, Australia, Benchmarking, Data envelopment analysis

## Abstract

**Background:**

Hospital-acquired complications (HACs) are costly and associated with adverse health outcomes, although they can be avoided. Administrative linkage health data have become more accessible and can be used to monitor and reduce HAC.

**Aims:**

This study aims to use linkage administrative data to benchmark the safety performance of hospitals and estimate the feasible magnitude that HAC can be reduced. We also identify risk factors associated with HACs, and estimate the effects of HACs on adverse health outcomes and hospital costs.

**Methods:**

This is a retrospective linkage cohort study. The cohort includes 371,040 inpatient multiple-day admissions of 83,025 cardiovascular disease patients admitted to public hospitals in 2010 with follow-ups until 2015.

Data envelopment analysis was applied to benchmark the patient safety performance of hospitals. Logistic regression was used to examine the odds of HAC and its effects on in-hospital mortality and 30-day readmission. Generalised linear models were used to identify the impacts of HACs on hospital costs and the length of hospital stay.

**Findings:**

On average, 9.3% of multiple-day hospital admissions were associated with HACs. The average HAC rate can be reduced by two percentage points if all hospitals achieve the safety record of best-practice hospitals. Old age and multiple comorbidities were major driving factors of HACs.

**Conclusions:**

Cardiovascular disease patients with HAC have a higher risk of death, stay longer in hospitals and incur higher health care costs. The average HAC rates can be reduced by two percentage points by learning from best-practice hospitals operating in the same region.

## Introduction

Hospital-acquired complications (HACs) are undesirable and unintended conditions that may arise during a hospitalised episode [1]. The literature has accumulated substantial evidence that HAC is associated with a significant increase in costs, length of hospital stay, hospital readmission and mortality [2,3]. In Australia, one in nine admissions involved a HAC, and the incidence increased to one in four admissions among overnight admissions in 2018 [4]. HACs were shown to be associated with more than 10 days of additional length of hospital stay per episode and four folds increase in hospital costs in Australia [5].

Fortunately, HACs are mostly preventable [6], and thus, reducing HACs has become one of the top priorities of healthcare stakeholders. HACs have been integrated into a funding model for Australian hospitals to take into account the risk profile of patients [7]. The HAC-integrated funding model provides fair compensation for hospitals with the capacity to take high-risk patients. However, the current risk model does not take into account the characteristics of hospitals. For example, regional hospitals often have poorer equipment or shortage of highly-skilled workforce. Hence, they are likely to have a higher HAC rate than those in major cities despite with a similar risk patient profile. Although the magnitude of HACs can be feasibly reduced, no study had taken into account how hospital characteristics can impact the extent of reduction in HACs.

The study fills the literature gap by benchmarking the patient safety performance of hospitals and estimate the extent to which HAC rates can be reduced, after controlling for hospital characteristics. The benchmark analysis is conducted using data envelopment analysis (DEA) method, which has been widely used to analyse and rank the operational performance of institutions in various industries such as agriculture [8], fishery [9], banking [10], education [11], tourism [12], traffic safety [13], and healthcare [14]. Our DEA-based benchmarking analysis will estimate the feasible range HAC rate can be reduced. Compare to the current HAC-risk adjustment model [7], our analysis take into account hospital characteristics such as size, location and types. We also investigate determinants of HACs and evaluate the effects of HACs on health outcomes and healthcare costs among cardiovascular patients.

## Methods

### Data

The main data set for this study includes 135,399 patients admitted to hospitals for CVD-related conditions in 2010 with follow-ups until the end of 2015. The total number of admission episodes during the study period was 1,790,807. The cohort was constructed by linking data from various sources, including the Hospital Admitted Patient Data Collection, Emergency Department Information System, National Hospital Cost Data Collection, Medical Benefits Schedule (MBS) and the Pharmaceutical Benefits Scheme (PBS). The MBS and PBS data include all medical services and pharmaceuticals dispensed in the community. Detailed data linkage process and description of variables are presented in the cohort protocol [15]. Ethics approval was granted by the Research Ethics Committee of Griffith University (2017/001).

HACs more commonly occur during overnight or multiple day admissions [16]; thus, we excluded admissions for same-day admissions, which account for 65% of total episodes. We also excluded admissions to private hospitals because detailed data on the characteristics and outcomes of private hospitals (e.g., bed capacity, locations, hospital costs) were not available. After applying all exclusion criteria, the study sample included 371,040 overnight admissions for 83,025 patients.

### Variable selection

We used the latest Australian guideline [17] to define HACs using ICD-10 diagnosis codes (see Supplementary material 1 for details). In particular, HACs were defined as having adverse health issues developed during the hospitalisation period. The adverse health issues were identified by subsequent diagnosis codes, which indicates that patients were not admitted to hospitals for reasons associated with these issues. Meanwhile, whether the issues occurred at hospitals was identified using the condition onset flag (COF). HAC was identified as an admission with a COF value of 1, which means “Condition with onset during the episode of admitted patient care”. Based on ICD-10 codes of subsequent diagnoses and COF flag, HACs were classified into 16 groups: 1) Pressure injury; 2) Falls resulting in fracture or intracranial injury; 3) Healthcare-associated infection; 4) Surgical complications requiring unplanned return to theatre; 5) Unplanned intensive care unit admission; 6) Respiratory complications; 7) Venous thromboembolism; 8) Renal failure; 9) Gastrointestinal bleeding; 10) Medication complications; 11) Delirium; 12) Incontinence; 13) Endocrine complications; 14) Cardiac complications; 15) Third and fourth degree perineal laceration during delivery; and 16) Neonatal birth trauma. The identifier of HAC 5 (Metadata Online Repository-METeOR identifier: 608995) is not currently available across coding system of Australian hospitals while our cohort does not include any maternity or pregnancy-related admission. Thus, HACs 5, 15 and 16 were excluded from this study. In the empirical analysis, we measured HAC as a dummy variable to represent an episode with at least one HAC.

The outcomes of interest were in-hospital mortality, 30-day readmission, length of stay and costs (including hospital costs and MBS/PBS expenditure). The selected covariates, based on the availability of data and the literature [7,18] included age, gender, Indigenous status, country of birth, Socio-Economic Indexes for Areas (SEIFA) quintiles, marital status, ICU usage, comorbidities, and location of hospitals. The SEIFA index represents the relative socio-economic advantage and disadvantage of areas in Australia [19]. The SEIFA index, constructed from various inputs such as income, education, and occupation, ranges from 0 to 1000; higher scores indicate higher socio-economic advantage.

### Statistical analyses

In the descriptive stage, the χ^2^ test was used to examine the variation in HAC rates across different genders, ethnicities and age groups. In the subsequent stage, logistic regressions were then applied to examine the effects of HAC on the odds of death in hospital and 30-day readmissions. Generalised linear models were used to examine the effects of HACs on costs and the length of hospital stay.

To benchmark the performance of hospitals in patient safety, a data envelopment analysis (DEA) [20] was conducted by constructing a production frontier that consists of best-practice hospitals [21]. DEA is a mathematical programming approach that estimates the ratio of weighted outputs and weighted inputs to examine the operational efficiency of decision-making units (e.g., hospitals). DEA has been applied widely to analyse operational efficiency in many fields, including healthcare [22]. In this study, we select the “safety” rate, which defines as 100%-HAC rate, as the single output that represents patient safety. Two inputs include the number of beds and a complexity index. The complexity index was constructed using the Charlson Comorbidity Index (CCI) and the rate of admission to an intensive care unit (ICU). The CCI was constructed from 12 comorbidities that were mostly correlated with the risk of one-year mortality and calculated using the algorithm developed Quan *et al.* [23]. The complexity index ranged from 0 (the least complex admission) to 1 (the most complex admission). In the benchmarking analysis, we derive a “simplicity index”, defined as 1-the complexity index, to represent a positive relationship between inputs and outputs required in the best-practice frontier.

There are two approaches to benchmark the safety performance of hospitals using DEA: input-oriented (i.e., minimise inputs while generating the same outputs); and output-oriented (maximise outputs while using the same inputs). In this study, we selected an output-oriented approach to benchmark hospitals because minimising inputs (i.e., number of beds and complexity of patient profiles) are often not feasible, particularly in public hospitals. Thus, the result of the benchmarking analysis is interpreted as how much a hospital can improve on patient safety rates compared to the best-practice hospitals with a similar input structure. The difference in the safety performance of best-practice hospitals and other hospitals is referred to as the “technical efficiency score”. In this study, the technical efficiency scores represent how much a hospital can improve its patient safety record compare to the best-practice hospitals with a similar input structure. Using the results of the benchmarking analysis and the location of hospitals, we can illustrate the spatial relationship between hospitals regarding patient safety in a peer network map, which is useful to monitor the safety performance and provide feedback for hospitals.

**Figure 1:**
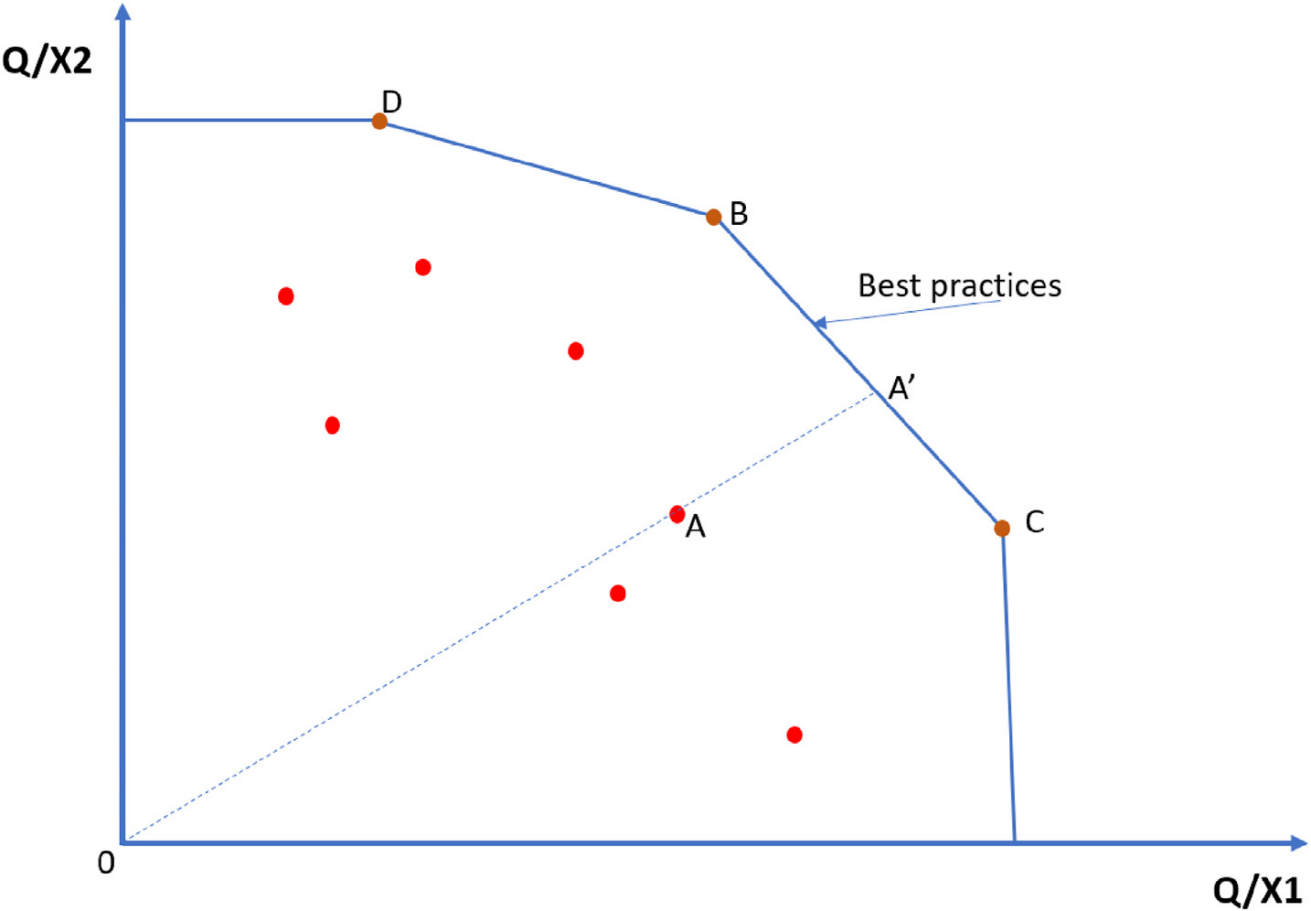
Benchmarking hospital performance using a production frontier.

The benchmarking can also be conducted at the state level, where all hospitals in the cohort are used to construct the best-practice frontier. Alternatively, the frontier can also be constructed using hospitals of the same regional group (e.g., major cities). The technical efficiency scores are often higher when using the group frontier, but the meta or overall frontier is more convenient to compare the performance of hospitals across regions (for technical details of estimation methods for group-frontier and meta-frontier using DEA, see Supplementary material 2). The mechanism for hospitals to improve their safety performance is illustrated in Figure 1, which represents a frontier constructed using one output (Q) and two inputs (X1, X2). Particularly, an inefficient hospital A can improve the patient safety performance by moving to A′ on the best practice frontier by learning from two efficient hospitals (best-practice hospitals) B and C that has a similar input-output structure. In this case, hospitals B and C are referred to as peers of hospital A. Details of how an inefficient hospital could learn from the best practice will require further investigation; the benchmarking analysis only identifies best practices and their relative peers. The ratio OA/OA′ represent the technical efficiency of hospital A. This hospital (A) can increase outputs from OA to OA′ by learning from hospitals B and C. Analyses were conducted using Stata 15.0 [24] and R 4.0 [25].

## Results

### Descriptive statistics

There was 34,340 out of 371,040 hospital admissions to public hospitals in our cohort involved at least one HAC, accounting for 9.3%. Also, HAC rates differed significantly by patient characteristics, admission characteristics and hospital locations (Table I). Given the well-known Indigenous health gap in Australia, it is somewhat surprising that Indigenous patients experienced lower HAC rates (7%) compared to non-indigenous counterparts (9.4%). One possible factor contributing to this finding is the relatively older age of non-indigenous patients in our cohort (71 years) compared with indigenous patients (55 years), as HAC rates increased substantially with age.

**Table I:** HAC rates by characteristics of patients, admissions and outcomes

Dummy variables	No (0)	Yes (1)
Sex (males=Yes)	0.092	0.093
Indigenous	0.094	0.070
*Age groups*		
Less than 45 years	0.096	0.061
45–54 years	0.095	0.068
55–64 years	0.095	0.081
65–74 years	0.092	0.095
75–84 years	0.087	0.108
85–94 years	0.090	0.109
95 years and older	0.092	0.114
*Marital status*		
Divorced/separated	0.093	0.091
Married	0.094	0.091
Never married	0.095	0.081
Widowed	0.089	0.108
*SEIFA quintiles*		
Q 1	0.096	0.083
Q 2	0.094	0.088
Q 3	0.090	0.101
Q 4	0.092	0.097
Q 5	0.091	0.099
Private insurance	0.093	0.089
ICU attendance	0.075	0.397
Acute episode	0.126	0.088
*Region of birth*		
America	0.092	0.097
Asia	0.092	0.096
Europe	0.091	0.101
Oceania	0.099	0.091
Africa	0.093	0.075
*Comorbidities*		
No comorbidity	0.098	0.066
One comorbidity	0.094	0.085
2+ comorbidities	0.074	0.100
*Hospital remoteness*		
Inner regional	0.097	0.075
Major cities	0.077	0.102
Outer regional	0.093	0.090
Remote	0.094	0.031
Very remote	0.094	0.025
	**Mean outcomes**	
	*No HAC*	*HAC*
Hospital costs ($)	7,755	32,621
Length of stay (days)	6.0	19.6
Die at hospital (%)	11.3	30.0
30-day readmission (%)	10.2	4.9

χ^2^ test show significant differences between groups except for sex.

Regarding marital status, widows experienced the highest average HAC rate of 10.8%. Again, age could be the main driving factor as those who outlived their husbands were an average of 82 years old, while the respective figure for the remainder was 67 years old. Socio-economic advantage, proxied by SEIFA quintile, have substantial effects on HAC rates despite the χ^2^ test showing significant between-group differences.

Patients with private hospital insurance had substantially lower HAC rates (8.9%) than those without private insurance (9.6%). Patients who were admitted to an ICU faced the highest HAC rates at 39.7%. In contrast, acute admission patients face much lower HAC rates (8.8%) compared to those of non-acute admissions (12.6%). Patients with more comorbidities, especially among those with two comorbidities or more, experienced significantly higher HAC rates (10.0%). The remoteness of hospitals was also significantly associated with HAC rates. Patients admitted to hospitals in major cities experienced the highest HAC rate (10.2%), while those admitted to hospitals in very remote locations experienced the lowest average HAC rate at 2.5%. One possible reason for higher HAC rates in hospitals located in major cities could be their greater capacity to admit patients with more complex health issues.

Costs and length of stay also differed significantly by HAC status. Particularly, a multiple-day admission without a HAC incurred $7,755 (Australian dollars, 2015 prices; A$1 ≈US$0.70 ≈ £0.49 using purchasing-power parity conversion tool available at https://eppi.ioe.ac.uk/costconversion/default.aspx) hospital costs compared to $32,621 for a multiple-day admission with a HAC. The average length of stay for a HAC admission was 19.6 days, while an admission without a HAC spent only six days in hospitals. HACs were also associated with almost three times the risk of death in hospital (11.3% vs 30.0%), but the risk of 30-day readmission was halved (4.9% vs 10.2%).

### HAC risk factors

Results of logistic regressions showed that indigenous patients faced no significant difference in HAC risk (Table II). However, age remained a significant determinant for HACs: patients aged 75 years and above faced at least double the risk of a HAC occurring. Patients who were admitted to ICU were ten times more likely to experience a HAC. Also, compared to those having no comorbidity, patients with two or more comorbidities had a 1.4-fold increased risk of experiencing a HAC. The hospital location also had a substantial effect on the odds of having a HAC. For example, compared to those admitted to hospitals in major cities, patients from hospitals in outer regional areas faced a 9% higher risk of HACs compared to those admitted to remote hospitals.

**Table II:** Determinants of HAC and health outcomes

Covariates	HAC	Die at hospital	30-day readmission
HAC		2.54 (2.43,2.66)	0.53 (0.51,0.56)
Sex (males=1)	0.96 (0.94,0.99)	1.20 (1.16,1.25)	1.06 (1.04,1.08)
Indigenous (Y=1)	1.01 (0.96,1.07)	1.00 (0.91,1.09)	1.50 (1.45,1.55)
*Age group (<45=base)*			
Age 45–54	1.09 (1.02,1.16)	1.54 (1.33,1.77)	0.89 (0.85,0.92)
Age 55–64	1.35 (1.27,1.43)	2.02 (1.78,2.30)	0.82 (0.79,0.85)
Age 65–74	1.70 (1.60,1.80)	2.84 (2.50,3.21)	0.77 (0.74,0.79)
Age 75–84	2.15 (2.03,2.28)	3.57 (3.15,4.04)	0.73 (0.71,0.76)
Age 85–94	2.40 (2.26,2.56)	5.33 (4.70,6.05)	0.71 (0.68,0.74)
Age 95+	2.65 (2.36,2.97)	8.46 (7.16,10.00)	0.60 (0.54,0.66)
*SEIFA quintiles (Q1=base)*			
Q 2	1.08 (1.04,1.12)	1.13 (1.07,1.19)	1.00 (0.97,1.02)
Q 3	1.21 (1.17,1.26)	1.06 (1.01,1.12)	0.96 (0.93,0.98)
Q 4	1.03 (0.99,1.07)	1.03 (0.97,1.10)	1.06 (1.03,1.09)
Q 5	0.94 (0.91,0.98)	1.06 (1.00,1.14)	1.13 (1.09,1.16)
*Marital status (divorced=base)*			
Married	1.01 (0.97,1.04)	1.21 (1.15,1.28)	0.92 (0.89,0.94)
Never married	1.05 (1.00,1.10)	1.10 (1.02,1.18)	0.98 (0.95,1.01)
Widowed	1.08 (1.04,1.13)	1.10 (1.03,1.17)	0.94 (0.91,0.97)
*Region of origin (America=base)*			
Asia	0.91 (0.79,1.03)	0.63 (0.52,0.77)	0.96 (0.86,1.07)
Europe	0.98 (0.88,1.08)	0.62 (0.54,0.71)	1.13 (1.04,1.22)
Oceania	1.02 (0.92,1.13)	0.67 (0.59,0.76)	1.15 (1.06,1.24)
Africa	0.76 (0.64,0.89)	0.60 (0.47,0.76)	1.31 (1.16,1.47)
Hospital insurance	0.95 (0.92,0.98)	0.98 (0.94,1.03)	1.08 (1.05,1.11)
Acute episode	0.57 (0.56,0.59)	0.16 (0.15,0.17)	7.63 (7.22,8.06)
ICU (Y=1)	10.07 (9.74,10.41)	4.48 (4.22,4.75)	0.61 (0.58,0.64)
*Comorbidity (No comorbidity=base)*			
Comorbidity=1	1.17 (1.11,1.23)	1.21 (1.12,1.32)	1.37 (1.32,1.42)
Comorbidity=2+	1.41 (1.36,1.47)	1.40 (1.31,1.49)	2.02 (1.96,2.07)
*Hospital remoteness (Major cities=base)*			
Inner Regional	0.85 (0.82,0.89)	0.91 (0.86,0.96)	1.14 (1.11,1.17)
Outer Regional	1.09 (1.05,1.13)	1.39 (1.32,1.47)	1.20 (1.17,1.23)
Remote	0.34 (0.30,0.40)	1.06 (0.91,1.22)	1.43 (1.35,1.52)
Very remote	0.47 (0.39,0.55)	1.05 (0.89,1.23)	0.95 (0.88,1.01)
Number of beds	1.00 (1.00,1.00)	1.00 (1.00,1.00)	1.00 (1.00,1.00)
Constant	0.04 (0.03,0.04)	0.04 (0.03,0.05)	0.02 (0.02,0.03)

*Note: Parameters are odd-ratios, 95% confidence intervals are in parentheses*.

After controlling for several confounding factors, HAC was significantly associated with the risk of in-hospital mortality and 30-day readmissions. In particular, the odds of death at the hospital was 2.5-fold higher for those with a HAC (95% CI: 2.4–2.7). However, the odds of being readmitted within 30 days from discharge was 47% lower for those with a HAC.

Other variables such as sex, ethnicity, age, and comorbidity were significant predictors of mortality risk. Particularly, males faced a 20% (16–25) higher in-hospital mortality risk and a 6% (4–8) higher risk of 30-day readmission. While Indigenous patients faced a 50% (45–55) greater risk of being readmitted within 30 days from discharge, there was no significant difference in their risk of dying at the hospital. Age was the most substantial determinant of in-hospital mortality risk. For example, patients aged 95 years or more were 8.5 times more likely to die at the hospital compared with those aged less than 45 years.

Patients admitted to ICU were 4.5 (4.2–4.8) fold more likely to die at the hospital, while their risk of readmission was 39% lower (36–42). Patients with one comorbidity were 1.2 (1.1–1.3) fold more likely to die at the hospital, and the respective figure for those with two or more comorbidities were 1.4 (1.3–1.5) folds. Patients with comorbidities were also more likely to be readmitted within 30 days from being discharged by 1.4–2.0 folds.

Compared to hospitals in major cities, patients admitted to hospitals in outer regional areas faced a 1.4 (1.3–1.5) fold increased mortality risk and 1.2 folds (1.17–1.23) increased risk of 30-day readmission. However, patients admitted to hospitals in very remote locations faced no significant difference in the risk of in-hospital mortality or 30-day readmission.

Compared to patients without HACs, those with HACs utilised more hospital resources, having three folds longer length of hospital stay, translating to three times higher hospital costs (Table III). Hospital costs and length of stay is shown to have an inverse U-shape relationship with age: increasing until the age 65–84 and then decreasing.

**Table III:** Effects of HACs on hospital costs and length of stay

Variables	Hospital costs	Length of stay
HAC	3.10 (3.06,3.14)	2.93 (2.90,2.97)
Sex (males=1)	0.98 (0.98,0.99)	0.97 (0.96,0.98)
Indigenous (Y=1)	0.96 (0.95,0.98)	0.91 (0.90,0.92)
*Age group (<45=base)*		
Age 45–54	0.98 (0.97,1.00)	0.99 (0.97,1.00)
Age 55–64	1.03 (1.01,1.05)	1.03 (1.01,1.04)
Age 65–74	1.08 (1.06,1.10)	1.10 (1.09,1.12)
Age 75–84	1.08 (1.06,1.10)	1.17 (1.15,1.18)
Age 85–94	1.05 (1.03,1.07)	1.22 (1.21,1.24)
Age 95+	1.02 (0.98,1.06)	1.18 (1.15,1.22)
*SEIFA quintiles (Q1=base)*		
Q 2	1.01 (1.00,1.02)	1.03 (1.02,1.03)
Q 3	1.02 (1.01,1.04)	1.00 (0.99,1.01)
Q 4	0.98 (0.97,0.99)	0.99 (0.98,1.00)
Q 5	0.89 (0.88,0.91)	0.99 (0.98,1.00)
*Marital status (divorced=base)*		
Married	1.02 (1.01,1.03)	0.96 (0.95,0.97)
Never married	1.04 (1.03,1.06)	1.07 (1.06,1.08)
Widowed	1.03 (1.01,1.04)	1.01 (1.00,1.02)
*Region of origin (America=base)*		
Asia	1.01 (0.96,1.05)	0.98 (0.94,1.01)
Europe	1.01 (0.98,1.05)	0.98 (0.96,1.01)
Oceania	1.01 (0.98,1.05)	1.00 (0.97,1.03)
Africa	0.95 (0.90,1.01)	0.89 (0.86,0.93)
Hospital insurance	0.88 (0.87,0.89)	0.95 (0.94,0.96)
Acute episode	0.67 (0.45,0.99)	0.44 (0.43,0.45)
ICU attendance (Y=1)	3.78 (3.72,3.84)	1.88 (1.85,1.90)
*Comorbidity (No comorbidity=base)*		
Comorbidity=1	1.08 (1.06,1.09)	1.15 (1.14,1.17)
Comorbidity=2+	1.23 (1.22,1.24)	1.30 (1.29,1.32)
*Hospital remoteness (Major cities=base)*		
Inner regional	0.95 (0.93,0.96)	1.00 (0.99,1.01)
Outer regional	1.01 (1.00,1.02)	1.12 (1.11,1.13)
Remote	1.22 (1.18,1.26)	0.99 (0.96,1.01)
Very remote	2.19 (2.10,2.28)	1.07 (1.04,1.10)
Number of beds	1.00 (1.00,1.00)	1.00 (1.00,1.00)
Constant	3669 (2472,5445)	4.48 (4.33,4.64)

*Note: Exponentiated parameters are presented; 95% confidence intervals are in parentheses*.

Among other covariates, hospital insurance and the severity of admissions were significant determinants of hospital costs and length of stay. Particularly, patients with private hospital insurance who incurred 12% lower hospital costs. Those admitted to ICU experienced 3.8 times higher hospital costs and a 1.9 times higher length of stay. Patients with comorbidities also faced 8–23% higher hospital costs and stayed longer at the hospital by 15–30%.

### Benchmarking hospital safety

Results from the data envelopment analysis showed that patient safety performance varied considerably by hospital location (Table IV). Using the regional frontier, hospitals located in major cities were the most efficient, with an average technical efficiency of 95.2% (i.e., HAC rates can be reduced by 4.8% by learning from best-practice hospitals in similar regions). In contrast, hospitals in outer regional areas can improve patient safety by 38.1%. The weighted average technical efficiency of all hospitals was 78.3%, suggesting that, on average, the HAC rate can be reduced by 21.7% if all hospitals can match the safety performance of best-practice hospitals in their region.

**Table IV:** Hospital safety performance by remoteness regions

Hospital location	Types of frontier	
	Regional frontier	State frontier
Major cities	95.23 (98.80, 91.66)	52.63 (59.66, 45.59)
Inner regional	84.16 (88.05, 80.27)	56.96 (59.85, 54.07)
Outer regional	61.90 (66.58, 57.22)	60.21 (64.33, 56.09)
Remote	86.45 (93.60, 79.31)	64.75 (72.65, 56.84)
Very remote	84.97 (91.40, 78.54)	59.52 (65.69, 53.34)
Average	78.28 (81.51, 75.04)	58.73 (61.09, 56.37)

*Note: 95% confidence intervals are in parentheses*.

As expected, benchmarking results using the state frontier show that hospitals can have more room for improvement with the average technical efficiency score of 58.7% (i.e., HAC rates can be reduced by 41.3% if all hospitals can match the safety records of the best-practice hospitals across the whole state). A closer examination reveals that the main factors contributing to this substantial gap between regional and state frontiers are two outstanding hospitals located in outer regional areas. This finding explains why the gap between state and regional frontiers is smallest among hospitals in outer regional areas (i.e., 61.9% vs. 60.2%). More detailed examination of these two best-practice hospitals are needed to determine whether their safety record can be replicable to all hospitals across the state. Thus, we now focus on discussing the results of the regional frontier.

**Figure 2:**
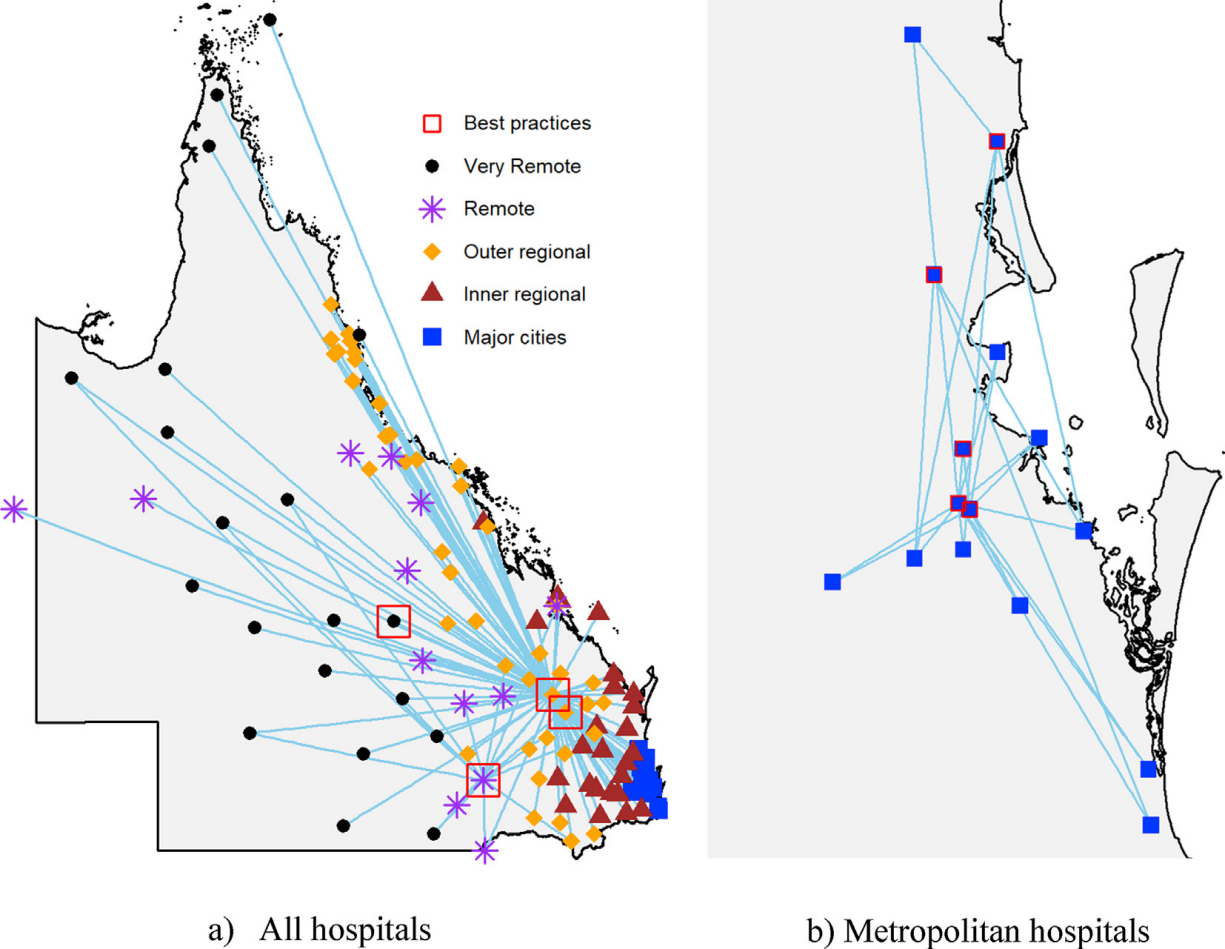
Hospital peer networks.

The peer network (Figure 2) shows that inefficient hospitals can improve their patient safety performance by learning from best-practice hospitals of the same region. We withhold the names of hospitals on the map to protect their identity. Also, benchmarking results of each hospital (e.g., technical efficiency scores, targeted inputs and outputs, peers and peer weights) are not presented on the map to avoid over-crowding. In practice, the benchmarking analysis will provide each hospital with a brief report on: 1) how efficient they are compared to the best practices (i.e. technical efficiency score); 2) what are the achievable target for inputs or outputs if they are 100% efficient (i.e., targets); 3) what are the best-practice hospitals that share similar input-output structure to them (i.e., peers); and 4) what are the relative importance of their peer hospitals (i.e., peer weights). The benchmarking results will provide hospitals with useful managerial information to improve the operational efficiency of hospitals. The peer network map illustrates that best-practice hospitals with a greater peer count (illustrated by the number of connections) are more influential on the safety performance of other hospitals. Best-practice hospitals with few connections could have a unique input-output structure making it difficult for other hospitals to learn.

## Discussion

HACs remain a public health concern as these are significantly associated with adverse health outcomes. This study confirmed that the risk of HAC was substantially higher for elderly patients, those admitted to ICU or those who had multiple comorbidities. The change in HAC risk was substantial among those aged 75 years, who are more than twice likely to have a HAC compared to those aged less than 45 years. This finding is consistent with the literature [26] that elderly patients often have comorbidities, dementia and frailty, which could result in a higher risk of HAC. However, admission to ICU was the strongest indicator of HAC risk with the odds ratio of more than 10. This finding was also in line with the literature [27] that patients in ICU was associated with higher risk of HAC. Overall, our findings suggest that efforts to reduce HAC should be focused on ICU units and vulnerable patients (e.g., old age and multiple comorbidities).

The risk of HAC was not significantly different between sexes and ethnic groups, after controlling for other characteristics of patients (e.g., marital status, and socio-economic status), characteristics of admission episodes (e.g., acute episode, ICU admission, and comorbidity), and hospital location. This finding suggests that the significance of the χ^2^ test for HAC rate by ethnicity groups reported in Table I could be due to differences in age and comorbidities because the test did not control for any covariates.

In line with the literature [[28], [29], [30]], this study found that HAC was associated with significantly higher hospital costs. The largest cost difference is observed for hospital cost, which shows that patients with HACs incurred higher hospital costs by 3.1 folds compared to those without a HAC. This finding is consistent with the difference in average hospital costs by HAC status being $24,800 ($32,621 vs. $7,755). Most of the hospital cost differences can be attributed to a 2.8-fold longer hospital stay (i.e., increase from 6.0 to 19.6 days). In addition, HACs were associated with a 2.5-fold increased risk of in-hospital death. The positive association between HAC and in-hospital mortality was consistent with studies in other countries, including the United States [31,32] and Europe [33,34].

Our findings that HAC is prevalent among the older age group is consistent with findings in the literature, which shows that age is a risk factor for HAC [2,35]. In addition, the higher risk of mortality and hospital costs estimated in this study are in line with previous estimates in the literature. For example, Glance *et al.*, [3] found that in the United States, trauma patients with HAC have 1.5–1.9-fold higher odds of death and incur 2–2.5 folds higher health care costs compared with those without HAC. Similarly, Chan *et al.* [36], estimated that the length of hospitalization among gynecologic cancer patients who had HAC was nine days longer than among those without HAC. They further found a cost difference, which was 2.87 times larger among patients with HAC. Overall, the high cost and high mortality risk associated with HAC suggest that reducing HAC could improve health outcomes and save costs. However, current strategies to reduce HAC focused only on patient characteristics [7] or incident reporting scores [37], which has been criticised for not comparing the performance of hospitals with similar characteristics [38,39]. The benchmarking analysis in this study addresses this issue by comparing the safety performance of hospitals with similar characteristics using the data envelopment analysis framework.

The benchmarking analysis revealed that hospitals could reduce HAC rates substantially by learning from best-practice hospitals in the same location and with similar characteristics. For example, Figure 2 illustrates that an inefficient hospital in major cities can learn from four best-practice hospitals to improve patient safety performance. On average, hospitals in the sample can reduce HAC rates by 21.7% or 7,452 episodes by learning from the experience of best practice hospitals. Since each HAC episode incurred an additional $24,800 in hospital costs, a reduction of 7,452 HAC episodes is translated to a cost-saving of $184.8 million.

## Conclusions

To the best of our knowledge, this is the first study to benchmark the patient safety performance of hospitals in Australia using DEA. This benchmarking analysis not only identifies the extent to which HAC can be reduced but provides useful managerial inputs (e.g., peers and targets) for hospitals to improve their patient safety performance. We also investigate the prevalence and determinants of HAC and its subsequent association with adverse health outcomes and healthcare costs. Our finding is consistent with the literature that old age and multi-morbidities were the main risk factors for HACs. However, managerial factors contribute to a potential reduction of the average HAC rate by two percentage points if lessons of the regional best practices can be shared. Considering the global increase in CVD related mortalities over the past two decades, it is necessary for policymakers, especially hospital managers, to put in place appropriate measures to minimize HACs.

Policymakers should consider adopting the benchmarking method used in this study to evaluate hospital safety performance to target setting and budget allocation. This method has been widely applied in healthcare [13] and other industries [8,[10], [11], [12],^14^] for performance benchmarking. The main advantage of our approach is that it can provide useful information such as peers (which best-practice hospitals are most relevant to learn from) and target (how much HAC and be reduced) for each hospital. However, a more detailed examination of best-practice hospitals will be required to promote experience sharing such that other hospitals can learn to improve their safety performance.

## CRediT authorship contribution statement

**Son Nghiem**: Conceptualization, Data curation, Formal analysis, Visualization, Writing original draft, Writing - review & editing, Investigation.

**Clifford Afoakwah**: Data curation, Writing - review & editing.

**Joshua Byrnes**: Investigation, clinical advice, Writing - review & editing.

**Paul Scuffham**: Writing – review & editing, supervision, project management.
