# Supplementary material for: Benchmarking hospital safety and identifying determinants of hospital-acquired complication: the case of Queensland cardiac linkage longitudinal cohort
Source: Infect Prev Pract. 2021 Dec 13;4(1):100198. doi: 10.1016/j.infpip.2021.100198 (PMC8717596; doi:10.1016/j.infpip.2021.100198)
Supplement: Multimedia component 2 [file mmc2.docx]

**Supplementary material 2: a technical note on meta-frontier, group frontier and data envelopment analysis**

*Meta-frontier and group-frontier*

A production frontier is defined the boundary optimal input-output combinations generated by best-practice hospitals. A group frontier is the production frontier of hospitals with similar production technology. A meta frontier is the frontier the covers all group frontier. This study constructs a meta-frontier and calculate the distance between the group frontiers and the meta-frontier using the concept of the *distance function*. In particular, such functions measure the distance from an actual observation to the technological frontier in an input-output space. To define the distance functions one must first define the production technology, which is the set of all feasible input-output combinations. A meta production technology *T* is defined as

$T=\left\{(x,y):x\text{ can produce }y\right\}$ (1)

where is a vector of inputs, and represents a vector of outputs. A meta-frontier is defined as the boundary of the output (or input) set in *T*. For example, the output set for an input vector ***x*** can be represented as:

$P(x)=\left\{y:(x,y)\in\text{ }T\right\}$ (2)

The boundary of this output set is referred to as the output “meta-frontier”. A meta output distance function (D_O_) is defined as follows:

$D_{O}(x,y)=\mathop{Max}\limits_{\varphi} \left\{\varphi\geq1,(x,\varphi y)\in P(x)\right\}$ (3)

Equation (3) is interpreted as the maximum amount of outputs that a hospital can produce from a given set of inputs. A hospital is fully efficient if D_o_(x,y)=1.

Similarly, the above concept can be applied to construct “group frontiers”. In particular, technology group *k* (*k*=1, 2, ..., *K*), output sets, and distance functions are defined, respectively, as:

$T^{k}=\left\{(x,y):x\text{ can produce }y \text{by all firms in group }k\right\}$ (4)

$P^{k}(x)=\left\{y:(x,y)\in\text{ }T^{k}\right\}$ (5)

$D^{k}_{O}(x,y)=\mathop{Max}\limits_{\varphi} \left\{\varphi\geq1,(x,\varphi y)\in P^{k}(x)\right\}$ (6)

As mentioned previously, a hospital is technically efficient if it has a distance function equal to unity. Therefore, the technical efficiency of a hospital at the meta-frontier and group frontier can be represented, respectively, by the distance functions:

$TE(x)=D_{O}(x,y)$ (7)

$TE^{k}(x)=D^{k}_{O}(x,y)$ (8)

The gap in technology between the meta-frontier and group frontier, is defined simply as the ratio of technical efficiencies between the two frontiers. This measure is referred to in the literature as the meta-technology ratio (MTR):

$MTR^{k}(x,y)=\frac{D_{O}(x,y)}{D^{k}_{O}(x,y)}=\frac{TE(x,y)}{TE^{k}(x,y)}.$ (9)

Because the meta-frontier envelops all group frontiers, the technical efficiency scores of the group frontiers are generally higher than those based on the meta-frontiers. This ratio is interpreted as the extent to which a hospital can further improve its production by moving from being efficient at the group frontier (a.k.a., ‘the best’) to being efficient at the meta-frontier (a.k.a. ‘best-of-the-best’). If a hospital achieves MTR=1, it is fully efficient in both frontiers, while MTR=0.8 implies that the hospital can further increase its outputs by 20 % by moving from the group frontier to the meta-frontier.

*Data envelopment analysis*

The calculation of the technical efficiency (TE) for any *i^th^* hospitals requires solving a linear programming problem to measure the distance from its input-output structure to the frontier as follows:

$D_{O}\{x_{i},y_{i}\}=Max\left\{\left.\varphi\geq1,(x, \varphi y\in p(x)\right|Y\lambda\geq y_{i},x_{i}\geq X\lambda\text{ }\lambda\geq0,\varphi\geq1\right\}\text{ }i=1, 2, ..., N,$ (10)

where φ is a scalar, λ is a vector of constants, representing the weights used to construct the weighted outputs/inputs achievable for the hospital to be fully efficient, X and Y are the matrix of inputs and outputs of all hospitals under investigation, respectively, and *x_i_* and *y_i_* are the vectors of inputs and outputs, respectively, of the *i*^th^ hospital.

The outcome of interest in DEA includes 1) efficiency score φ represent the magnitude in which a hospital can increase more outputs (e.g., patients discharged) using the current level of inputs; 2) vectors of weights represent the relative importance of each peer that the inefficient hospitals can learn from to improve their operational efficiency. From the weight and peers, DEA also generate target inputs and outputs for hospitals if they are fully efficient.
